# Ovarian Cancer, Cancer Stem Cells and Current Treatment Strategies: A Potential Role of Magmas in the Current Treatment Methods

**DOI:** 10.3390/cells9030719

**Published:** 2020-03-14

**Authors:** Nuzhat Ahmed, Elif Kadife, Ali Raza, Mary Short, Paul T. Jubinsky, George Kannourakis

**Affiliations:** 1 Fiona Elsey Cancer Research Institute, Ballarat, Victoria 3353, Australia; elif@fecri.org.au (E.K.); alir@students.federation.edu.au (A.R.); George@fecri.org.au (G.K.); 2 Federation University Australia, Ballarat, Victoria 3353, Australia; 3 Centre for Reproductive Health, Hudson Institute of Medical Research, Monash University, Clayton, Victoria 3168, Australia; 4 Department of Obstetrics and Gyanecology, University of Melbourne, Parkville, Victoria 3050, Australia; 5 Developmental and Molecular Biology, Albert Einstein College of Medicine, Bronx, NY 10461, USA; mary.short@einstein.yu.edu (M.S.); paul.jubinsky@einsteinmed.org (P.T.J.)

**Keywords:** ovarian cancer, treatment, magmas

## Abstract

Epithelial ovarian cancer (EOC) constitutes 90% of ovarian cancers (OC) and is the eighth most common cause of cancer-related death in women. The cancer histologically and genetically is very complex having a high degree of tumour heterogeneity. The pathogenic variability in OC causes significant impediments in effectively treating patients, resulting in a dismal prognosis. Disease progression is predominantly influenced by the peritoneal tumour microenvironment rather than properties of the tumor and is the major contributor to prognosis. Standard treatment of OC patients consists of debulking surgery, followed by chemotherapy, which in most cases end in recurrent chemoresistant disease. This review discusses the different origins of high-grade serous ovarian cancer (HGSOC), the major sub-type of EOC. Tumour heterogeneity, genetic/epigenetic changes, and cancer stem cells (CSC) in facilitating HGSOC progression and their contribution in the circumvention of therapy treatments are included. Several new treatment strategies are discussed including our preliminary proof of concept study describing the role of mitochondria-associated granulocyte macrophage colony-stimulating factor signaling protein (Magmas) in HGSOC and its unique potential role in chemotherapy-resistant disease.

## 1. Introduction

Most ovarian cancers (OC) are high grade malignancies involving one or both ovaries, with substantial metastatic potential [1,2]. Among malignant gynaecologic diseases, OC has the second highest incidence in Western countries after cervical cancer, and the third highest prevalence in Asian countries [3]. Prognostic factors for advanced OC include age, post treatment residual tumour volume, tumour histology, presence or absence of ascites, and serum CA-125 levels [4]. A combination of taxane and platinum-based therapies has been the standard treatment of OC for more than four decades. Five year patient survival and overall survival has essentially remained unchanged because of the lack of new effective therapy and persists in the 30–40% range [5]. Recurrent OC remains a major challenge since there is >80% patient mortality within 5 years [6]. The failure of chemotherapy treatment in OC is a likely result of tumour heterogeneity, lack of targetable effects on tumour microenvironment, and the likely presence of pre-existing or acquired chemoresistant cancer cells or cancer stem cells (CSCs) [7,8].

### Different Origins of OC

The biology of OC has been hard to comprehend, as these tumour cells appear to closely resemble any of the cells normally present in the ovary [2]. Histologically OC can be classified into five sub-types: high-grade serous carcinoma (HGSOC), low-grade serous carcinoma (LGSOC), mucinous carcinoma (MC), endometrioid carcinoma (EC) and clear cell carcinoma (CCC). For HGSOC, no reliable precursor lesion was histologically recognized until 16–17 years ago [9,10]. Risk-reducing salpingo-oophorectomy allowed identification of serous fallopian tube intraepithelial carcinoma (STIC) as the origin of HGSOC in tumors containing BRCA1 and BRCA2 mutations [10]. Subsequent studies have shown that HGSOC and STIC have a common ‘p53 signature’ (mutation in p53) [11,12]. The ‘p53 signature’ was further described as a DNA damage response activated by serine/threonine kinases, ataxia-telangiectasia mutations (ATM), and ATM-and Rad3-Related kinase (ATR) at the sites of DNA double strand breaks, which likely originates in STICs [13,14]. Human fallopian tube cells transformed in vitro produce tumours that are morphologically and genetically similar to HGSOC [15,16,17]. Genetically engineered mouse OC models containing mutations commonly found in HGSOC such as *p53*, *PTEN*, *BRCA1*, *BRCA2*, and *Rb1* in tubal secretory cells have shown the phenotype of STIC formation and emergence of HGSOC [18,19,20]. Clinical studies have confirmed that the majority of women who undertook salpingo-oophorectomy due to the presence of *BRCA1, 2* mutations had STICs in the fallopian tube. Most of these carcinomas were present in the fimbria (finger-like projections at the end of the fallopian tube) of the fallopian tube, suggesting that the fimbria may be the origin of HGSOC [13,21,22].

Ovarian surface epithelium (OSE) appears to be the origin of other subtypes of HGSOC [23,24]. One theory of the etiology of OC suggests that physical trauma provoked during ovulation results in increased inflammatory cytokines and reactive oxygen species (ROS), that initiates DNA damage in OSE [25]. Accumulation of these events over time may result in malignant transformation. Other studies show that damaged DNA repair is hindered in OSE trapped in cortical inclusion cysts (CICs) [23,25]. Some morphological, histological and epidemiological studies indicate that CICs have tumorigenic potential [25,26,27]. The epithelium lining of CICs can consist of ciliated or secretory tubal cells, flat OSE-type, or a mixture of tubular and OSE cells [2,28]. However, ovarian CICs consisting of ciliated or secretory tubal cells are more likely to give rise to HGSOC, while OSE-type cells mostly give rise to LGSOC [2,29]. It has also been postulated that the detachment of fimbrial secretory cells with a ‘p53 signature’ adjacent to OSE may also be the origin of HGSOC [2]. These studies indicate the different anatomical origins of HGSOC, which complicates the pathology and molecular characteristics of this cancer [2,30].

Current classification has simplified OC into two major groups: type 1 tumours are low-grade with reduced growth rate and are mainly restricted to the ovary at diagnosis; and type 2 tumours are high-grade rapidly proliferating, which spread to organs outside the ovary, specifically to the peritoneum and the omentum [31]. Type 2 tumours are large masses of multinucleated cells, which have a greater disease volume throughout the peritoneal cavity compared to type 1 tumour. These tumours follow a stepwise progression from a benign precursor lesion to a malignant state [32]. They have accelerated mitotic index and an active DNA damage repair mechanisms (DDR) with effective ‘p53 signature’ [13,31]. These tumours may display gene amplification and over expression of the *HER2/neu* and *AKT2* oncogenes [30]. On the other hand, mutations in *KRAS*, *BRAF*, *PTEN* and *Wnt/β-catenin* are common in type 1 tumours [33]. Nearly 90% of ovarian tumours are type 2 HGSOC, while only 5–10% of serous sub-type are type 1 LGSOC tumours [30]. Type 1 tumours also consist of major histological subtypes of OC such as endometrioid (cells resembling the endometrium), mucinous (cells resembling endocervical glands), and clear cell carcinoma (clear cells containing glycogen). Genetic studies have demonstrated type-1 tumours to group independently of type-2 tumours, implicating that these two groups have a different genetic basis [24].

## 2. Heterogeneity in OC

In addition to different origins of HGSOC, OC progression is further made complex by tumour heterogeneity, which can be classified as either inter-tumour, or intra-tumour heterogeneity [7]. Inter-tumour heterogeneity occurs if the genotypic and phenotypic variation exists between multiple tumour cells from one patient, for example the primary lesion of OC patients may be different in genotype and phenotype from the tumours of distant omental metastasis [34]. On the other hand, intra-tumour heterogeneity occurs if genotypic and phenotypic variation occurs within the same tumour of primary lesion or distant metastases [7,35]. Both inter- and intra-tumour heterogeneity occurs in OC as well as in most cancers and is the primary cause of disease progression and importantly therapeutic resistance and relapse [7]. Tumour heterogeneity in OC can arise from genetic or epigenetic changes as well as clonal expansion of cells driven by these changes [7].

### 2.1. Genetic Changes

The genetic changes in OC results from chromosome instability (CIN) [7,36]. High levels of CIN have been reported in HGSOC [37]. CIN may result from the stimulus arising from the peritoneal microenvironment. Peritoneal fluid shear force [7,38] and defects in DNA repair [7,39] are able to induce CIN in ovarian and fallopian tube cells Germline mutations in *BRCA1*, *BRCA2*, *BRCA*-Fanconi anaemia associated mutations (*RAD51C*, *RAD51D*, *BRIPI* and *BARDI*) and mismatched repair genes (*MSH2*, *MLH1*, *MSH6*, *PMS2* and *EPCAM*) and *STK11* [7,40] have all been implicated. 

### 2.2. Epigenetic Changes

Epigenetic changes also contribute to heterogeneity in OC [7,41]. Epigenetic changes modify gene expression but do not affect the original DNA sequence. The main factors controlling epigenetic changes in OC include gene-specific hypermethylation of DNA, posttranslational modification of histones (histone acetylation) or mRNA, such as microRNAs (miRNA), or global hypomethylation [7]. This review focuses on gene-specific hypermethylation, which usually involves abnormal hypermethylation of CpG sites of promoter regions of coding genes, which silence the transcription of the relevant gene in daughter cells following cell division [7]. A family of DNA methyltransferases (DNMTs) facilitates this process. The levels of DNMTs are enhanced in cancer cells compared to normal cells [42,43]. Many tumour suppressor genes are hypermethylated in OC (*SLIT2*, *PTEN*, *OPCML*, *RASSF1A*, *p16, MLH1*, *E-cadherin* and *APC*) [7,44]. *BRCA1* mutations occur in hereditary breast cancer and OC, but sporadic *BRCA1* promoter methylation is observed in 15–30% of OC [7,45]. Hypermethylation of CpG islands of *OPCML*, *RASSF1A*, *MINT25*, *DCR1*, *HIC1*, *PTEN*, and *BRCA1* have been reported in early-stages of OC progression [46]. Hypermethylation of *BRCA1* results in the loss of DNA repair mechanisms, which contributes to CIN and tumour heterogeneity in OC [7]. In addition, hypermethylation of cell cycle control genes *PTEN* and *RASS1A* contributes to increased tumourigenesis in OC [7,47]. Hypermethylation of several pro-apoptotic genes including *DAPK, LOT1,* PAR4 and *TMS1/ACS* have been reported in response to chemotherapy treatment in OC cells [7]. These genes are involved in chemoresistance mechanisms and contribute to relapse in OC. However, methylation of genes (*BRCA1, GSTP1, and MGMT*) implicated in DNA repair and drug detoxification processes were associated with a better response to chemotherapy [7,44]. 

Besides hypermethylation, global DNA hypomethylation due to decreased levels of 5-methyl-deoxycytidine (5mdC) has been shown in cancer [48]. Global DNA hypomethylation usually occurs at the repetitive elements of the genome, including the interspersed retrotransposon *LINE-1* [48], which makes up approximately 17% of human genome. In that context, hypomethylation of LINE-1 has been reported in OC [48]. In addition, it has been shown that hypomethylation is a common event in STICs, the precursor lesion of HGSOC [48]. Global DNA hypomethylation has also been reported in OC [48], and has been linked with advanced-stage disease and reduced overall and disease-free survival [48]. Significantly enhanced levels of hypomethylation of satellite DNA have been observed in OC patients but not in patients with benign and borderline tumours, and this hypomethylation correlates with advanced-stage disease and poor prognosis [48,49]. A genome-wide methylation profiling of blood cells from healthy controls and age-matched untreated OC patients identified CpG methylation of 2714 cancer-related genes, of which 56% were hypomethylated [50]. 

## 3. Ovarian Cancer Stem Cells

Cellular heterogeneity associated with genetic and epigenetic changes in tumours are linked with the initiation and expansion of CSCs, a population of cells within the tumour, which initiate tumour growth through self-renewal and differentiation processes [51,52]. These cells are more adaptive to the changing tumour microenvironment and tend to be more resistant to treatment. CSCs are highly plastic and a few numbers of isolated CSCs have been shown to be responsible for tumorgenesis and are more chemotherapy and radiation resistant due to their dormant state and a high expression of drug efflux pumps [53,54]. As a result, CSCs are able to adapt to different tumour microenvironments and survive due to their efficient DNA repair mechanisms and their capacity to evade host immune surveillance [55,56,57]. These inherent properties of CSCs suggest an active role in tumour relapse and emphasize the need to develop effective targeted therapies to improve clinical outcomes in patients [51,52,53,54,55,56,57].

Stem cells have been identified in ovaries and fallopian tubes [58,59]. These cells express proteins including aldehyde dehydrogenase family 1 A2 (ALDH1A2), homeobox protein NANOG, LIM homeobox protein 9 (LHX9) and frizzled-related protein 1 (SRFP1) and have been observed in OSE, CIC and fimbria [2,58,59,60,61,62]. In addition, stem-like cells were identified in the OSE of adult mouse ovary [63]. These dormant cells have enhanced proliferation during the estrous cycle and possess protective mechanisms against cytotoxic agents. However, their capacity for long-term self-renewal and differentiation could not be established [63]. Recently an area between the OSE, mesothelium and oviductal epithelium of the mouse ovary, known as the hilum region, was noted as a stem cell niche [64]. A discrete population of cells in this region was shown to express stem cell markers [aldehyde dehydrogenase (ALDH)-1, leucine-rich repeat containing G-protein-coupled receptor (LGR)-5, lymphoid enhancer-binding factor (LEF)-1, prominin-like protein-1 (CD133), cytokeratin 6B (CK6B)]. These cells were prone to transformation after inactivation of p53 and retinoblastoma protein (Rb)1, the pathways that are generally inactive in advanced HGSOC [64].

The first CSCs were identified from a single cell in the ascites of an OC patient, which had the ability to serially generate tumours over several generations in mice [65]. In OC, CSCs have been identified by the expression of cell surface and non-surface markers originating from ovarian tumours and ascites. For example, distinct expression of cell surface markers [i.e., CD44, epithelial cell adhesion molecule (EpCAM), CD133, CD117, Thy1, CD24] [55,66,67,68] and non-surface markers (aldehyde dehydrogenase) [69] for CSCs have been reported in OC. These CSCs have demonstrated potential of a ‘CSC trait’ (ability to self-renew, resistance to therapy, develop tumours in very small numbers ~100 cells), however, their significance in a clinical setting is yet to be established.

A distinct population of cells [known as the ‘side population’ (SP)] isolated from ovarian tumours and cultured ovarian cancer cell lines have the ability to efflux the DNA binding dyes [70]. SP cells possess typical CSC properties in relation to in vivo tumourigenicity and are more resistant to chemotherapy than the more differentiated malignant cells [71]. However, heterogeneity of cells within the side populations has been observed with groups of cells maintaining more expression of some stem cell markers such as Oct4, CD117, and CD44 than others within the same population. In that context, CSCs expressing distinct markers may have characteristic inherent properties, which potentially could provide selective advantages to different groups of CSCs. Cancer patients may contain multiple pools of CSCs in their tumours and these pools may vary significantly between different patients making targeted therapy against CSCs difficult. Using two ovarian cultured cell line models grown under non-adherent conditions, and treated with cisplatin and paclitaxel, multiple CSC pools expressing CD133^+^/CD117^-^, CD133^+^/CD117^+^ and CD133^-^/CD117^+^ were obtained by flow cytometry [72]. Functional analyses of these phenotypes indicated that both CD133 and CD117 contributed different growth or functional advantage to the relapsed tumour. While CD117^+^ cells provided survival advantage over CD117^-^ cells via the AKT pathway, CD133^+^ cells provided metastatic and adhesion advantage over CD133^-^ cells [73,74]. Although the expression of CSC markers varies significantly between tumours from the same or different patients, it is not clear, whether these different CSC sub-clones originate from the same progenitor and if that has any effect on prognosis as well as response to therapy in patients. The plasticity of CSCs, which affects differentiation of cancer cells into different lineages further complicates the identification of CSC populations [75,76]. The process of CSC plasticity is also manifested by the processes of the epithelial-mesenchymal transition (EMT) and the reverse mesenchymal-epithelial transition (MET), which facilitate the migration of OC cells to distant sites and initiates future establishment of tumours at secondary sites [76,77]. Induction of EMT is also seen in OC cells after treatment with platinum-based (cisplatin) chemotherapy that results in the enrichment of residual chemoresistant cells which are capable of initiating aggressive relapse in animal models [77,78]. However, it is uncertain whether chemotherapy-induced CSCs have similar genotypes/phenotypes to native CSCs in tumours. It would be useful to understand the role of each putative and drug-induced CSC population in patient prognosis, disease recurrence, and response to therapy.

Signal transduction pathways have been shown to maintain the CSC phenotype in OC [79]. Several pathways, including those involving PI3/PTEN/AKT [80], JAK2/STAT3 [81,82,83], NFkB [84,85], Notch [86,87], Wnt [86,87], and Hedgehog [86,88] support the CSC phenotype and promote tumourigensis and therapy resistance in OC. Inhibiting these pathways in ovarian cell lines and OC animal models suppresses CSC numbers and makes these cells sensitive to chemotherapies [79].

## 4. Metastases and Tumour Microenvironment in OC

Metastases in HGSOC occur through several distinct routes, which include, transcoelomic, hematogenous and lymphogenous spread [89]. The spread of OC occurs primarily through transcoelomic route, which involves metastasis in the peritoneal cavity and the surrounding peritoneal organs [90,91]. Metastasizing OC single cells, multicellular aggregates, and spheroids seed the peritoneal cavity mesothelial layer and the peritoneal cavity organs [1]. These cells invade the extracellular matrix and underlying stroma, which contains activated fibroblasts, endothelial cells lining the blood vessels, innate and adaptive immune cells and the lymphatics. The microenvironment supplies inflammatory factors enabling malignant cell and endothelial cell proliferation, and inhibitors to immune function [92]. Anoikis-resistant OC cells shed from the tumours as single cells or sheets, survive in peritoneal tumour microenvironment as spheroids resulting in further dissemination [93]. 

Disseminating OC cells express a variety of membranous receptors and adhesion molecules such as CD44, L1 cell adhesion molecule (LICAM), CD24, integrins, CX3CL1, and CD133 to attach to mesothelial cells [66,94]. Membrane type-1 matrix metalloproteinase (MT1-MMP), a major transmembrane interstitial collagenase appears vital for the release of cells from the tumour [94,95]. MT1-MMP activation is mediated by epidermal growth factor receptor, Src and wingless (Wnt) signaling pathways [94,95]. In contrast, OC cells adhesion to sub-mesothelial collagen matrix, occurs via α2β1 and α3β1 integrins [96,97]. A population of OC cells expressing keratin 14 appear to initiate the invasion process [98,99]. 

Adipose tissue is an important regulator of OC tumour progression [100]. The primary OC mass is in close proximity to ovarian fat pads and adipose deposits and nearby fat layers in the mesentery and diaphragmatic peritoneum [101]. Adiponectin, interleukin-6, interleukin-8, C-X-C motif chemokine ligand 1 and others derived from omental adipocytes support the survival and dissemination of tumor cells to regions with increased levels of inflammatory cells [102,103,104]. OC cells regulate adipocyte fatty acid metabolism and the transfer of lipids from adipocytes to tumour cells, which enhances beta fatty acid oxidation and tumour cell proliferation [104,105,106]. 

In addition to transceolomic dissemination, distant metastasis in OC results from hematogeneous spread to thyroid, bone, skin, heart, breast, kidney and brain seen in small numbers of OC patients [107,108,109]. Abdominal, pelvic and thoracic lymph node mediated metastases have been observed in FIGO Stage III and IV OC patients [96]. The presence of distant metastasis is rarely the direct cause of mortality, but is associated with aggressive disease and a poor prognosis [108,109].

## 5. Magmas (Mitochondrial Associated Granulocyte Macrophage Colony Stimulating Factor Signaling Molecule)

Mitochondrial associated granulocyte macrophage colony stimulating factor signaling molecule (Magmas) was identified as a GM-CSF inducible gene in a hematopoietic cell line [110]. Magmas (yeast ortholog is called Pam16) is an essential, highly conserved, mitochondrial protein which is necessary for the viability of all eukaryotic cells [110,111,112]. It belongs to the type IV class of J-proteins and regulates the ATPase activity of the inner membrane protein import motor by inhibiting DnaJC19 [113]. Loss of Magmas activity impairs protein import and oxidative phosphorylation resulting in increases in reactive oxygen species, cell cycle arrest, and loss of viability [113,114,115].

The expression of Magmas protein by immunohistochemistry in *Mus musculus* varies during development but does not strictly correlate to the differentiation state of the cell [111]. During murine embryogenesis elevated expression of Magmas has been noted in heart, cervical ganglion, notochord, nasal mucosa and liver [111]. In adult mouse tissues, elevated levels of Magmas expression is observed in several tissues including muscle, acinar pancreas, the perirenal and proximal tubules of the kidney, and intestinal lining. Blood vessels and fibrous tissue in the ovarian stroma were the only ovarian cells with detectable Magmas expression, while only the endometrial mucosal cells showed some staining in the uterus [111].

Magmas mRNA expression was found to be elevated in murine ACTH secreting pituitary adenomas compared to normal pituitary tissue by differential display [116]. A screening of 64 human pituitary adenomas showed that two thirds of these adenomas had elevated Magmas message levels compared to normal, control pituitary tissue. [116]. Decreasing Magmas mRNA expression by 80% with shRNA in the ACTH-secreting murine AtT-20D16v-F2 cell line reduced DNA synthesis, and increased G1-S phase arrest [116]. In addition, while Magmas shRNA treated cells expressed similar basal caspase 3/7 activity and DNA fragmentation compared to controls, both caspase 3/7 activity and DNA fragmentation was significantly increased compared to controls in the presence of 5% DMSO, (used to induce apoptosis) [116]. Consistent with this observation, Magmas overexpression in the rat pituitary adenoma GH4C1 cell line inhibited apoptosis in staurosporin treated cells compared to controls [117]. 

Several studies have demonstrated the relevance of Magmas in human disease. An evaluation of Magmas protein expression in human prostate carcinoma pretreatment biopsy samples by immunohistochemistry suggests that Magmas has a role in this disease. Malignant prostate glandular tissue was shown to have variable elevated Magmas protein expression compared to the adjacent normal glandular tissue, which had weak expression [118]. Approximately 50% of the high grade prostate adenomas had weak Magmas expression while the remaining samples showed moderate to high expression of Magmas. The increased Magmas expression observed in malignant tissues resulted from enhanced protein expression of Magmas protein and not to an increase in the mitochondrial content of the cells [118].

The effects of Magmas on cell viability appear to be mediated by reactive oxygen species (ROS) levels [119]. In this study, overexpression of Magmas in the PC-3 human prostate cancer cell line was shown to reduce the level of ROS and prevent ROS-facilitated activation of caspases 3/7 essential for apoptotic cell death. Magmas affects the ROS production by boosting the electron transport chain (ETC) activity and function of two major antioxidant enzymes; magnesium dependent superoxide dismutase (MnSOD) and a glutathione peroxidase (GPx), which facilitate ROS scavenging [119]. Conversely, downregulation of Magmas in human cell lines and yeast enhanced the ROS level in cells and made these cells more vulnerable to ROS-associated apoptosis. All of these metabolic effects of Magmas were independent of its role in protein import [119]. This study suggests a mechanism by which Magmas overexpression may be of key importance in the initiation and maintenance of human malignancies.

Magmas overexpression has also been reported in human gliobastoma (GBM) resection samples and in tumors derived from the syngenetic subcutaneous injection of the GL261 murine glioblastoma cell line [120]. A small molecule Magmas inhibitor, BT#9 (compound 9) [121], decreased proliferation in human GBM cell lines (D-54 MG, U-251 MG), murine embryonal stem cell lines (1123 Mes, 83 Mes) and a human glioblastoma stem cell line (HuPuP01). The inhibitor also reduced cell migration and invasion, increased apoptosis, and reduced oxidative phosphorylation in human GBM cell lines [120]. These results are consistent with the previously cited findings that reduction of Magmas activity in cancer is a sound treatment strategy, including those having a malignant stem cell subpopulation.

Finally, a temperature sensitive mutation in Magmas is responsible for an early severe skeletal dysplasia (spondylometaphyseal dysplasia) [122]. In addition to the considerable skeletal changes, these patients had developmental delay and died from cardiomyopathy or pulmonary insufficiency, demonstrating an important role for Magmas during human development.

### Magmas Expression in Ovarian Tumours, OC Cell Lines and in Mice Xenografts Before and after Chemotherapy Treatment, and the Effect of Magmas Inhibition on a Chemotherapy-Treated Ovarian Cancer Cell Line: A Proof of Concept Data

**Figure 1:**
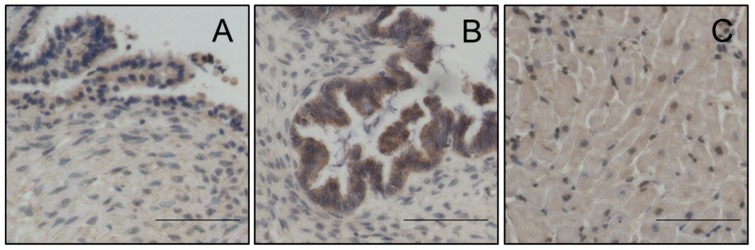
Magmas expression is higher in HGSOC than benign serous tumours. Representative immunohistochemistry images of Magmas in a (**A**) benign serous tumour and a (**B**) HGSOC. Benign (n = 8) and HGSOC (n = 17) were obtained from patients diagnosed with OC undertaking surgery at The Royal Women’s Hospital after obtaining written consent under protocols approved by the Human Research and Ethics Committee (Ethics approval #09/09) of the Royal Women’s Hospital, Melbourne, Australia. Benign tumours were from patients undertaking total abdominal hysterectomy or bilateral salpingo-oophorectomy due to pre-diagnosed medical conditions. Immunohistochemistry on paraffin-embedded human serous ovarian tumours was performed as described previously [83]. (**C**) Negative controls were prepared by incubating tissue sections without the primary antibody followed by the secondary antibody. Stained slides were scanned at 20× magnification using Leica EVOS FL Auto 2 microscope (Thermo Fisher). Sections were assessed microscopically for positive DAB staining. Scale bar = 100 µm, Magnification is 20×.

**Figure 2:**
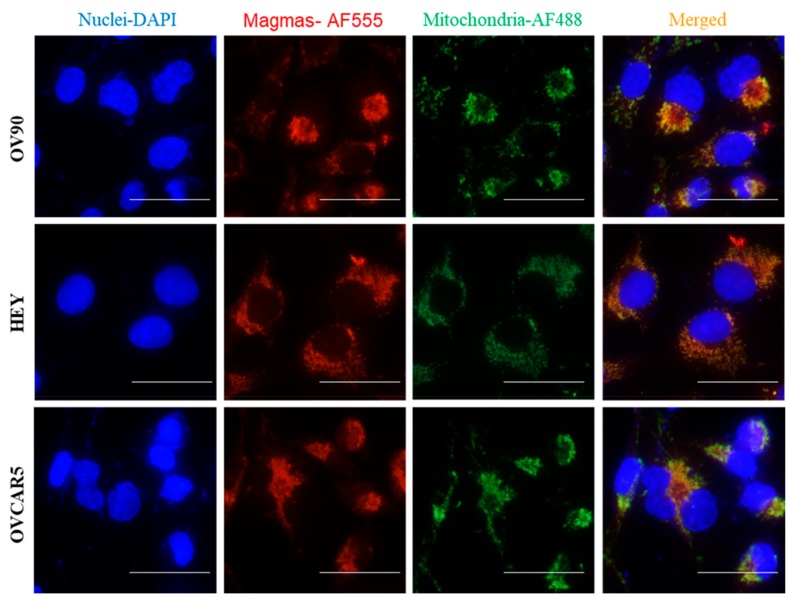
Co-localization of Magmas with the mitochondrial fluorescent dye marker AF488 in OV90, HEY and OVCAR5 human ovarian cancer cell lines. OV90, HEY and OVCAR5 cells were fixed with 4% paraformaldehyde, and probed with Magmas targeting primary antibody [83] and co-stained with mitochondria specific primary followed by respective secondary antibodies (AF555, AF488, Life Technologies). DAPI (4′,6-diamidino-2-phenylindole) (Invitrogen, Carlsbad, USA) was used to stain cellular nuclei. Fluorescence imaging was performed using Leica EVOS FL Auto 2 microscope. Scale bar = 75 µm, Magnification is 40×.

Magmas expression was evaluated by immunohistochemistry (IHC) using anti-Magmas antibody on eight benign serous tumours and seventeen HGSOC. There was significantly more expression of Magmas in HGSOC compared to benign tumours (Figure 1). The expected mitochondrial localization of Magmas in human OC (Figure 2) was confirmed by co-localization with mitochondrial dye marker AF488 by fluorescent microscopy in the human OC cell lines HEY, OVCAR5 and OV90.

**Figure 3:**
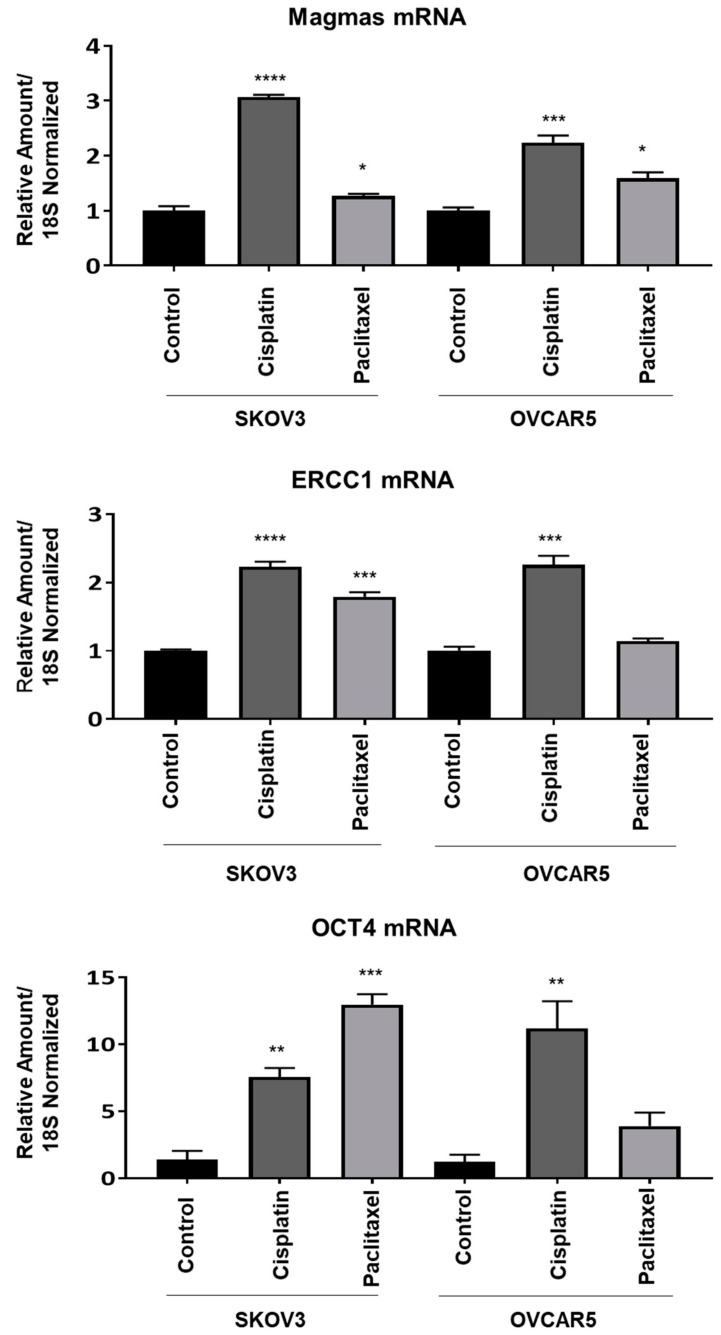
The mRNA expression of Magmas, ERCC1 and OCT4 in SKOV3 and OVCAR5 ovarian cancer cell lines. Magmas, ERCC1 and OCT4 mRNA expression in SKOV3 and OVCAR5 cells were performed after treatment with IC_50_ doses of cisplatin or paclitaxel as described previously [83,123]. The relative expression of gene of interest was normalized to housekeeping 18S gene. Data are shown as the mean of +SEM (n = 3). Significance between the groups was deduced by One-way ANOVA and is indicated by * *p* > 0.05, ** *p* > 0.01, *** *p* < 0.001, **** *p* < 0.0001.

**Figure 4:**
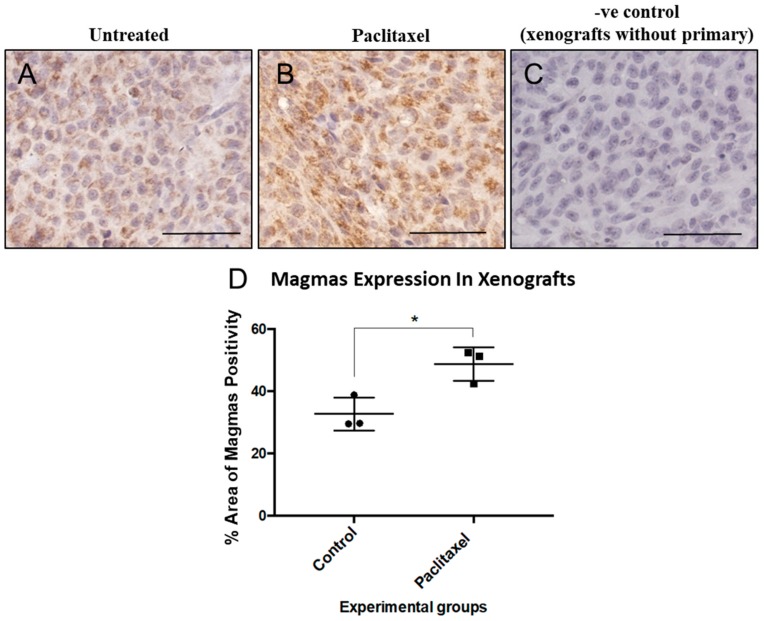
Magmas expression was induced in xenografts treated with chemotherapy. Animal experiment was performed in strict accordance with the recommendation in the Guide for the Care and Use of Laboratory Animals of the National Health and Medical Research Council of Australia. The experimental protocol was approved by the University of Melbourne Animal Ethics Committee (Project-1413207.1). Female Balb/c nu/nu mice (age 6–8 weeks) were injected intraperitoneally (i.p) with 5 × 10^6^ ovarian cancer HEY cells. Paclitaxel treatment started 19 days after cancer cell induction and mice received 2 rounds of paclitaxel treatment once a week at 15 mg/kg dose before being culled at the same time as control mice. Immunohistochemistry on paraffin-embedded tumours was performed as described previously [57,81,82,83]. Magmas expression was significantly lower in (**A**) untreated tumours compared to those that were (**B**) treated with paclitaxel. (**C**) Negative controls were prepared by incubating tissue sections without the primary antibody followed by the secondary antibody. Stained slides were scanned at 20× magnification using Leica EVOS FL Auto 2 microscope (Thermo Fisher). (**D**) Sections were assessed microscopically and quantitatively for positive DAB staining using Imagej FIJI. Scale bar = 100 µm. Significance is indicated by * *p* < 0.05 by T-test.

Studies were performed to determine the effects that standard chemotherapy has on Magmas, excision repair complementation complex protein 1 (ERCC1), and Oct4 mRNA expression. All were significantly elevated in human ovarian cancer SKOV3 and OVCAR5 cell lines exposed to carboplatin or paclitaxel (Figure 3). The increase in Oct4, a stem cell transcription factor required in maintaining pluripotency, may reflect either an induction of Oct4 message or a selection for more immature cancer cells with higher Oct4 mRNA. Magmas protein expression was correspondingly elevated when an ovarian cancer cell line (HEY) xenograft [57,81,82,83] was treated in vivo with paclitaxel (Figure 4).

**Figure 5:**
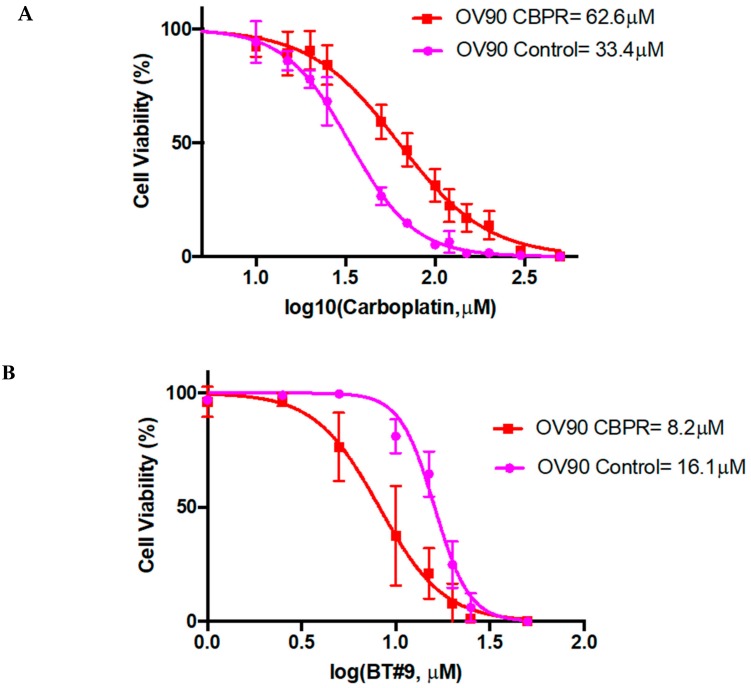
Sensitivity of OV90 parental (control) and carboplatin resistant (CBPR) ovarian cancer cells to carboplatin and Magmas inhibitor BT#9. OV90 parental control and OV90 CBPR cells (5 × 10^5^ cells/well) were seeded and treated with varying concentrations of carboplatin and BT#9 drugs (carboplatin 72 h or BT#9 48 h). Cell viability was checked by WST-1 assay kit was used according to manufacturer’s instructions. (**A**) IC_50_ values of OV90 control cells and OV90 CBPR cells in response to Carboplatin; (**B**) IC_50_ values of OV90 control cells and OV90 CBPR cells in response to BT#9. Assays were conducted three times in triplicate.

The most interesting finding is that a small molecule Magmas inhibitor (BT#9) reduced the viability of an OV90 carboplatin resistant cell line significantly more than the parental OV90 cell line (Figure 5A–B). In contrast, the Magmas inhibitor was considerably less cytotoxic than carboplatin on the carboplatin sensitive cells.

These data suggest that Magmas has a major role in chemotherapy resistance in OC cells, providing a compelling ‘proof of concept’ that Magmas inhibition could have a beneficial effect in the treatment of chemotherapy resistant OC patients. Our previous studies have shown that OC cells after treatment with chemotherapy are enriched in a population of cells having CSC-like characteristics [76,78,81,82,83]. In this scenario, the expression of Magmas may decrease ROS production in CSC cells making them resistant to cytotoxic stress resulting from chemotherapy. Experiments demonstrating a suppressive effect of BT#9 on CSC expansion would provide additional evidence supporting the benefits of reducing Magmas activity in OC patients.

## 6. Ovarian Cancer Treatment Strategies 

### 6.1. Current Treatment

In the last 30 years, a five-year survival rate of all cancers has improved by 20% [124]. However, the 5 year survival rates for OC patients has changed very little for that period even in socially, economically and technologically advanced countries like United States and Canada, and remains at ~40% compared to 85% for breast cancer patients [125]. Worldwide 239,000 new cases of OC are diagnosed each year and 152,000 deaths occur making OC the seventh common cancer and the eighth common cause of cancer-related death in women [124,125]. Although 90% of early-stage disease has a survival rate of >10 years, the majority of women diagnosed with stage III/IV disease die of the recurrent disease within 5 years [126]. Primary debulking surgery followed by combination therapy of platinum-taxane has been the main treatment for decades [127,128]. However, well-conducted clinical trials in the past decade have resulted in more structured care for OC patients [129,130,131]. 

Sequential clinical trials have indicated that the best survival outcome for OC patients can be achieved if there is no residual disease (R0) after surgical resection of the tumour [132]. Post-surgery standard practice takes into account histology, stage, genomic profile and the extent of residual disease [125]. Most OC patients respond favorably to the initial choice of platinum and/or taxane therapy but recurrence will occur within few months [133]. Different lines of targeted therapy are initiated after first recurrence. Over the past decade, the introduction of concurrent or sequential bevacizumab and poly (ADP-ribose) polymerase (PARP) inhibitors in addition to treatment by first line combination therapy has achieved a significant progress in terms of improved progression-free survival and less toxicity in platinum resistant patients, who develop recurrence within the first six months of front line platinum and taxane therapies [128]. Other antiangiogenic agents, such as the tyrosine kinase inhibitors pazopanib, sorafenib, sunitinib, cediranib and AMG386 have also been assessed but these have not been adapted into clinical practice due to toxicity and/or cost of licensing [125,134,135]. In case of platinum-sensitive patients, who develop recurrence after the initial 6 months of front line therapy, challenge with double platinum-based chemotherapy has been recommended. In addition, non-platinum agents such as liposomal doxorubicin, weekly paclitaxel, gemcitabine and other agents have been incorporated [125]. However, each of these combinations ultimately results in sequential recurrence. Hence, search for more targeted therapy with promising novel agents are currently under clinical trials.

Patients with germline BRCA1/BRCA2 mutations or other homologous recombination deficiency (HRDs) (which constitute 50% of HGSOCs, mostly are platinum sensitive patients) are sensitive to PARP inhibitors [136,137]. This offers a rationale for using platinum-based chemotherapy in combination with PARP inhibitors in HGSOCs. As a result, few PARP inhibitors (olaparib, niraparib and rucaparib) have been introduced in clinic by the US food and drug administration (FDA) as a maintenance therapy for HGSOC patients [137]. Recent clinical trials selected platinum sensitive BRCA1/BRCA2 mutated patients for olaparib, niraparib and rucaparib as maintenance monotherapy after front line platinum therapy [137,138,139,140,141,142,143,144]. These trials showed consistent improvement in progression free survival of platinum-sensitive recurrent patients with a range of efficacy on patients with mutated BRCA1/BRCA2 as well as those with HRD and those with no HRD. However, as HRDs exist in most OC patients, subsequent trials have tested PARP inhibitors in platinum resistant patients and have shown encouraging results. Toxicity studies and evaluation of quality of life demonstrated that the PARP inhibitors mentioned above delay recurrence and assist patients to sustain a superior quality of life [140,141]. In recent clinical trials, a combination of PARP (Niraparib) and angiogenesis inhibitor (Bevacizumab) or veliparib in combination with chemotherapy have shown superior results in patients with or without HRD and those with or without BRCA mutations [145,146,147,148]. However, some patients do develop resistance to PARP inhibitors and there are ongoing genomic studies to determine the key genomic factors, which distinguishes long-term responders to patients who develop resistance (short-term responders) [125]. In addition, in vitro and in vivo studies in OC have shown that PARPi induces an enrichment of CD133^+^ and CD117^+^ CSCs, which may contribute to therapeutic resistance [149]. These CSCs undergo cell-cycle arrest in G2-M phase, accrue **γ**H2AX, RAD15 and DMC1 foci and demonstrate accelerated DNA repair mechanism [149]. Hence, previously sustained and/or PARPi-induced DNA repair process can contribute to innate resistance in response to PARPi treatment. Other studies in glioblastoma multiforme [150] and colorectal cancer [151] have shown that inclusion of PARPi to radiotherapy or chemotherapy enhances the effectiveness of chemotherapy by sensitizing CSCs to chemo/radiotherapy. This occurs due to the reduction in the level of CSC self-renewal and DNA repair processes [150]. Some of the current and ongoing clinical trials involving PARPi and other agents is described in Table 1.

### 6.2. Pathway to Precision Medicine

The term ‘personalized medicine’ or ‘precision medicine’ are used for groups of patients or an individual patient on whom medical decisions for tailored therapeutic interventions are implemented based on their genomic pattern which determines their unique susceptibility to a disease. This enables targeted therapy to facilitate better treatment outcome. As cancer leaves a significant mark on a patient’s genome, genomic profiling of a tumour is considered as the driving force for personalized medicine. In 2017, FDA approved cancer treatments for patients carrying specific genetic signatures in their tumours (The Scientist, July 15, 2019) (https://www.fda.gov/news-events/press-announcements/fda-approves-first-cancer-treatment-any-solid-tumor-specific-genetic-feature). Currently, several genetic tests are in practice to meet the therapeutic needs of a diverse range of cancers (https://www.fda.gov/medical-devices/vitro-diagnostics/list-cleared-or-approved-companion-diagnostic-devices-vitro-and-imaging-tools) (The Scientist, July 15, 2019). However, in many cases, the genome fingerprint does not always present adequate indicators for targetable genes. This is due to our lack of understanding of the mechanisms, which controls genes. Even, in the scenario of diseases where mutation in one gene is the cause of a disease, accurate diagnosis founded only on the genetic information is not always satisfactory [152,153]. In the case of cancer, where mutation burden in the tumour cells is high and the cells are continuously undergoing multiple mutations, it is more difficult to predict a genotype to stratify a therapeutic strategy [153]. Moreover, genomic profiling of tumours can be a long process. As decisions for the treatment of OC patients should ideally start within the first few days after surgery, genomic profiling data may not be attainable at the start of a treatment plan. The data from the Cancer Genome Atlas (TCGA) and the International Cancer Genome Consortium (ICGC) that methodically profiled hundreds of HGSOCs is publically available [154]. Gene expression studies have also classified HGSOC into four prognostic subtypes: differentiated, immunoreactive, mesenchymal and proliferative [155]. Of these, the immunoreactive subtype is associated with best overall survival while the mesenchymal subtype is associated with worst overall survival [155]. A recent study has reported a potential clinical utility of using a 10-gene signature regulated by TGFβ1 in advanced OC patients with poor prognosis [156]. Although these studies classified signatures in HGSOCs for better or poor survival, their clinical implementation is difficult and remains untested in clinical practice. Hence, other potential new avenues that hold prospect for exploration in the era of personalized medicine in HGSOC patients are essential. There is a consensus in the scientific community that strategies, which target factors such as tumour heterogeneity, genomic instability, overexpression of oncogenes, and loss of tumour suppressor genes, up regulated signaling pathways, the regulatory mechanisms between cancer cells, CSCs and the tumour microenvironment, may identify potential targetable sites for precision medicine. Some of the evolving therapies, which are being explored in the era of precision treatment for OC patients, are discussed below.

### 6.3. Targeting Folate Receptor (FR)

Targeting of FR is evolving as a new therapy for the treatment of OC patients [125]. The normal ovarian tissue does not express FR but 70% of OCs and 80% of recurrent OCs express FR [157]. Hence, exploiting FR as a target for the delivery of antibody drug conjugate consisting of an anti-FRα antibody linked to a tubulin-disrupting maytansinoid DM4 drug, a potent antimitotic agent has shown promise in Phase 1 and 2 trials in 37 women who had moderate to high expression of FRα expression [158]. These trials showed tangible responses in patients with median progression-free survival of 6.7 months [159]. These encouraging results have led to the design of Phase 3 trial in primary peritoneal or fallopian tube cancer and in platinum resistant OC patients [160]. 

### 6.4. Immunotherapy

In a recent meta-analysis of 10 studies involving 1815 OC patients, the infiltration of both CD3^+^ and CD8^+^ tumour-associated lymphocytes (TILs) were linked with better overall survival, but CD8^+^ TILs were associated with a more positive outcome [161]. In addition, analysis of CD8^+^ TILs in the tumour epithelium of 24,650 patients revealed that the median survival for patients without TILs was 2.8 years, while with low, moderate or high TILs survival was enhanced to 3 years, 3.8 years and 5.1 years respectively [162]. Other studies have confirmed the presence of CD8^+^ and CD20^+^ TILs to correlate positively with overall survival of OC patients [163]. In addition, 73.9% patients with pre-existing TILs had complete response after debulking surgery followed by platinum-based chemotherapy, while complete response was achieved in only 11.89% without TILs [163,164]. These findings suggest that OC are ‘immunogenic tumours’ that could produce substantial anti-tumour immune responses to inhibit progression and facilitate therapy outcomes. However, there are factors in the tumour microenvironment that can impair the activity of TILs, thereby facilitate cancer progression. On the other hand, a negative impact on tumour progression by specific T cell subsets of the immune system in the ovarian tumour microenvironment has been reported. These include T cells expressing cytotoxic T lymphocyte-associated antigen 4 (CTLA-4), glucocorticoid-induced TNF receptor family-related protein (GITR), or CD8^+^CD28^-^Tregs, exhausted CD8^+^T cells expressing immune checkpoint inhibitory molecules programmed cell death-1 (PD-1) or lymphocyte activation gene-3 (*LAG-3, CD223*) [165,166]. In addition, most ovarian tumours overexpress PD-L1 and indolamine 2,3-dioxygenase (IDO1) and/or secrete cytokines like VEGF and TGFβ which enhances the proliferation of regulatory cells (Tregs) and facilitate their action thus promoting T cell suppressive microenvironment and functional exhaustion [167,168]. In addition, downregulation or loss of expression of major histocompatibility complexes (MHC)-I and II on the tumour cells leads to decreased cytotoxic T lymphocyte action [169]. As a result, recent cancer immunotherapies (checkpoint inhibitors) have shown low response rates in OC patients [170]. Hence, currently there are no approved immune therapies for OC patients [170]. However, new approaches are introduced to enhance the efficacy of checkpoint inhibitors, such as combination of anti-CTLA-4 and anti-PD-1 [171], or a combination of checkpoint inhibitors with chemotherapy, such as PEGylated liposomal doxorubicin or weekly paclitaxel [172], or combination with epigenetic agents [173], antiangiogenic agents such as bevacizumab in combination with PARP inhibitor niraparib (ANITA) [172,173]. A combination of epigenetic therapy (entinostat) with PD-L1 inhibitor avelumab is currently in progress in recurrent OC patients (NCT02915523) [44,173]. These trials may offer additional information into clinical response in OC patients in response to combination of epigenetic and immunotherapy. In addition, clinical trials are recently investigating the introduction of chimeric antigen receptor (CAR) that target mesothelin, a membrane glycoprotein overexpressed on OC cells [124]. Few clinical trials based on immunotherapy and other agents are described in Table 1.

### 6.5. Metronomic Chemotherapy

An alternative to maximum tolerated dose (MTD) chemotherapy is metronomic chemotherapy or low-dense dose chemotherapy, which involves prolonged administration of low, equally spaced out doses of chemotherapeutic drugs, which not only provides therapeutic efficacy but also has lower toxicity. This is an efficient and inexpensive way to treat different cancers, including OC [174]. Metronomic chemotherapy provides several advantages compared to standard MTD protocol. This includes a decrease in tumour vascularization by targeting the active tumour stroma which is a source of angiogenesis regulator (antiangiogenic response) [175,176], lower therapeutic resistance of CSCs [177] and most importantly sufficient stimulation and recruitment of natural killer cells, dendritic cells, macrophages and cytotoxic T cells without their depletion [177,178]. In addition, metronomic chemotherapy can also result in the decrease of the number of immunosuppressive Tregs and myeloid suppressor cells further improving patient’s prognosis [178]. 

Metronomic chemotherapy can circumvent the enrichment of CSCs in the tumour microenvironment, which is facilitated by MTD doses of chemotherapy [55,80,81,82,83,177]. MTD regimen when administered to tumours induced selection of therapy-resistant clones by elimination of sensitive clones, making the residual recurrent tumour more aggressive and resistant to therapy. In addition, MTD- induced apoptotic and necrotic cells release intracellular stores of nutrients, which therapy-resistant cancer cells use to repopulate the tumour rapidly [176,177]. Moreover, traditional MTD chemotherapy regimen target rapidly dividing cancer cells, having a sparing action on slow dividing CSC population [176,177]. Furthermore, checkpoint regulators such as Rad17 and chk1/2 are upregulated by CSCs after MTD treatment, which dampens the surveillance of the host immune system [177]. However, metronomic chemotherapy coupled to immune checkpoint inhibitors may increase immune cell’s access to CSCs in the tumours facilitating their eradication [177]. Hence, a combination of metronomic chemotherapy and immune checkpoint inhibitors may provide an opportunity to target therapy-resistant cells, including CSCs, resulting in improved long-term outcomes and patients quality of life for difficult to treat cancers.

Several clinical trials using metronomic doses of chemotherapy in heavily treated recurrent patients or in elderly frail OC patients have been tried each showing longer progression free and overall survival compared to MTD regimen [178,179,180]. However, there are no approved metronomic treatment protocols for OC patients. There is however, a growing interest for further studies in metronomic approaches in this tumour type.

### 6.6. Nanoparticle Drug Delivery

Another aspect, which could aid precision medicine, is the use of drug delivery devices, which can be customized, for optimal dose delivery to the intended organs or tissues at the specified time [153]. Engineering trendy biomaterials for drug delivery systems, which can be adapted for an individual’s specific needs, is an appealing strategy for devising precision medicine [153]. Nanoparticle-based drug delivery systems accumulate in tumours much more than in normal tissues due to leaky cell-cell junctions in tumours [181,182]. As cytotoxic chemotherapies provide a narrow therapeutic period, nanoparticle formulation can enhance the pharmacokinetic features of the chemotherapy and facilitate effective targeting of tumours via the enhanced permeability and retention effects [182,183]. As a result, a low dose of nanoparticle-formulated chemotherapy may be as effective as a MTD regimen currently used in standard practice, thus alleviating the toxicity and other side effects discussed in Section 6.5. The nanoparticle vehicles that are presently in clinical practice or are being investigated for anticancer treatments include liposomes, protein-drug nanoparticles, polymeric micelles and dendrimers [183]. Clinical data have shown different pharmacokinetics and toxicity profiles of these formulations compared to their parent drugs [183]. Numerous nanoparticle drug formulations are currently in development and evaluation stages in early and late-phase clinical trials [183]. These nanoparticles are loaded with ligands such as antibodies, peptides, aptamers for targeting various plasma membrane associated antigens on tumours, thus allowing formulations to be customized for patients [183,184].

Nanoparticle-based drug delivery system is still in its preclinical development stage in OC [185]. A recent study has shown the use of hyaluronic acid-labeled poly (d,l-lactide-co-glycolide) nanoparticle (HA-PLGA-NP) formulated with paclitaxel and focal adhesion kinase siRNA as an effective delivery system against chemoresistant OC xenograft models [185]. This strategy was designed to target CD44 positive tumour cells abundantly found in ovarian tumours and to knock down focal adhesion kinase, which is upregulated in chemoresistant OC cells [186]. Although this study provided a novel mechanism for focal adhesion kinase knockdown using a siRNA-formulated nanoparticle-based system to tackle chemoresistance in OC, other signaling pathways involved in chemoresistance mechanisms can be explored. Recently, we have shown that lipid-based cubosome formulated paclitaxel provides superior results in terms of greater reduction in tumour burden and increased progression-free survival in a mouse model of OC [184]. 

### 6.7. Patient- Derived Tumour Organoid Modelling

The last two decades have seen the use of cancer cell lines and patient-derived xenograft (PDX) models as a tool for screening and assessing drug responses in cancer [187,188]. However, it has been reported repeatedly that the histological and genetic features of cancer cell lines are significantly different from the tumours from which they were derived [187,188]. As a result, many drugs that showed significant efficacy in a significant proportion of cancer-specific cell lines failed in clinical trials. PDX models on the other hand mimic the biological characteristics of its primary tumours but their application is limited as they are not only expensive but are labor and time consuming [189]. In addition, human tumours in mouse also evolve and change characteristics under the pressure of host physiology. Hence, there is a need to develop newer models that can surpass the shortcomings of cell lines and PDX models. 

Organoids are three-dimensional aggregates of tumour cells that grow in gel or on special low attachment plates that the cells cannot attach to [190,191,192]. Tumour organoids can be initiated from surgical tumour sections or from needle biopsies, or even from circulating tumour cells obtained from needle biopsies [193,194]. Organoids can be salvaged from frozen reserves and expanded in culture for several months without loss of proliferative capacity [192,195]. In the last few years, different research groups have developed organoids from patient-derived normal and tumour tissues, including breast, pancreas, prostate, liver and brain, and have shown that these models are able to reiterate histological, morphological, phenotypical and genetic aspects of host tumours [196,197]. Besides the phenotypic and genotyping profiling, the gene mutation spectrum of patient’s tumours was also retained in patient-derived organoids [191,193,198,199,200,201]. These organoids can be expanded long-term in culture without any loss of original characteristics of the derivative primary tissues and can be used for high-throughput drug screening [202]. In addition, few recent studies have shown that molecular profiling of patient-derived organoids is able to predict the accuracy on whether a patient would respond to specific drugs [191,203]. Hence, patient-derived organoids are the best platform for personalized medicine where individual organoid can preserve the histological, biological and genomic profile of a patient, and each organoid can imitate a patient as in a clinical trial [192,203]. Hence, each individual patient will potentially get an opportunity to receive precise treatment relying on the response of his or her organoids. As the heterogeneity of cancer, subtypes are retained within the organoids, each organoid’s response to drugs mimics with the real patient’s response in clinic. A good source of organoids derived from cancer patients can be initiated within a short period to form a ‘Living Biobank’ and effective drug screening can happen to stratify treatment strategies [192,194,203]. These biobanks essentially can act as the depositories of patient-derived tumour organoids of various tissue origins, which will enable advance research and treatment strategies [203]. In addition, when taking biopsies from patients with cancer normal tissue biopsies can also be acquired without much inconvenience to the patients. These normal and cancer organoids can be used to determine the therapeutic response of drugs on healthy tissue versus tumour from the same patient to alleviate toxicity issues and facilitate the design of best treatment outcomes [190,191,192,193,194,195,196,197,198,199,200]. Moreover, organoids can be used for gene editing using CRISPR/Cas9 knock out techniques to identify key driver mutations requisite for cancer development and progression [204,205,206]. A most recent study has succeed in generating tumour reactive T cell population by co-culturing peripheral blood lymphocytes with tumour organoids from mismatch repair-deficient colorectal cancer and non-small cell lung cancer [195]. This particular proof of concept study has a great potential in assessing the sensitivity of tumour cells to T-cell mediated cancer cell killing at the level of an individual patient. This can form the screening platform of personalized immunotherapy for patients with different cancer sub-types. Hence, patient-derived organoids hold the key for the development of a platform for personalized cancer treatment.

### 6.8. Ovarian Cancer Patient-Derived Organoid Model

A handful of papers have recently shown the development of OC organoids. A recent elegant study demonstrated the development of 56 organoid lines from 32 patients representing non-malignant benign tumours, mucinous tumours, clear cell carcinomas, endometroid tumours, LGSOC and HGSOCs [207]. These OC organoids recapitulated the histological and genomic features of the tumour lesions from which they were derived and could be manipulated genetically by genes requisite for HGSOC progression [207]. These organoids could be expanded long-term beyond 30 passages, conserved, and successfully recovered after preservation (85% success rate) [207]. The organoids were used for drug screening assays and were shown to recapitulate the different OC subtype responses to gold standard platinum therapy [207]. In addition, these OC organoids could be xenografted further facilitating drug sensitivity assays in vivo [207].

The strength of OC organoid cultures against 2D monolayer cultured cell lines was tested using an automated microscopic assay that could distinguish between cell death and inhibition of proliferation by various drugs [208]. This study showed that following treatment with standard chemotherapeutic drugs, the effects on organoids were more diverse and was linked to patient’s genome alterations in relation to drug sensitivity and DNA repair deficiency. However, this was undetectable in monolayer cultures suggesting that screening of OC organoids with relevant drugs would increase the accuracy to tailored cancer treatments to a more personalized care. A most recent study interrogated the DNA repair inhibitor response on 33 organoid cultures from 22 HGSOC patients with defects in homologous recombination (HR) and replication fork protection [209]. They were able to show that irrespective of the mutational status of the DNA repair gene, a functional defect in HR in the HGSOC organoids correlated with PARP inhibitor sensitivity [209]. However, functional defect in replication fork protection correlated with carboplatin resistance while checkpoint 1 and serine/threonine specific protein kinase for DNA damage sensing ATR sensitivity related to PARP inhibitor resistant tumours [209]. In another study, a novel method of developing OC organoids by seeding the cells around the rim of wells (mini-rings) instead of within a lump of gel was recently demonstrated [210]. These tumour organoids had a clear center that allowed efficient pipetting of drugs and other diluents. Using this system, two concentrations of 240 different kinase inhibitors were tested on ovarian organoids derived from four patients, three with OC and one with tumour of the peritoneum [210]. This robust mini-ring approach allowed testing of small number of cells without the need for expansion in vitro and was relatively quick where the results were available within a week after surgery. This technique if automated and scaled to 384 well plates can in future facilitate high throughput screening of multiple drugs on organoids making it ideal for personalized medicine.

### 6.9. CSC-Based Therapy

The presence of a high level of CSCs in tumours carries a bad prognosis and is associated with poor overall survival, high incidence of disseminated cancer and recurrence, indicating a strong negative prognostic indicator for CSCs containing tumours [211]. Some of the defining characteristics of CSCs such as cell surface markers, altered metabolism, altered signaling pathways; regulatory factors in the tumour microenvironment sustaining and enriching CSC volumes have the potential to be targeted for efficient eradication of CSCs [211,212]. In that context, targeting the cell surface receptors overexpressed in OC cells has shown credibility in preclinical models. The cell surface receptors and intracellular CSC markers that can be targeted for personalized therapies are discussed as follows:

CD44: It is a cell surface transmembrane hyaluronic acid receptor highly expressed in primary and metastatic OC cells. CD44 is involved in cell-matrix interactions that affect cell growth and motility [213]. CD44 positive cells express high levels of stem cell markers such as Oct4 and nestin, have enhanced NFκβ activity and high expression of IL1β, IL6, and IL8 [54]. These characteristics associate negative prognosis in patients with high CD44 expressing tumours. ONCOFIDTM-s, a conjugate of hyaluron and chemotherapy agent SN38 has shown strong anti-proliferative effect in vitro on OC cells [214]. Hyaluronic acid-labeled poly (d,l-lactide-co-glycolide) nanoparticle (HA-PLGA-NP) formulated with paclitaxel and focal adhesion kinase siRNA as a selective delivery system has shown strong efficacy against chemoresistant OC in preclinical mouse models [185].

Aldehyde dehydrogenase 1 (ALDH1): Approximately 50% of OC patients have high ALDH1 expression and that correlates with poor overall survival [215]. Some recent studies have shown dual expression of ALDH1 with CD44 (ALDH1^+^CD44^+^) or CD133 (ALDH1^+^CD133^+^) in primary ovarian tumour samples and linked that with reduced disease-free and overall survival in OC patients [215,216,217,218]. ALDH1 promotes tumour cell survival by reducing the in vivo cytotoxic damage induced by the oxidized aldehydes [218,219]. The enzyme also detoxifies residual cytotoxic materials left after chemotherapy treatment conferring resistance to ALDH1^+^ cells [218,219]. Few recent studies have explored ALDH as an attractive antigenic target for inducing ALDH specific immune responses [220]. Immuno-dominant epitopes derived from ALDH were used to generate cytotoxic effector CD8^+^T after treating them with autologous dendritic cells (DC) to target specifically ALDH^+^ cancer cells [221,222]. This provides a strong foundation of future utilization of ALDH1 as a CSC-associated antigenic target to develop immunotherapies in OC. In this context, Oct4 reactive CD4^+^ and CD8^+^ T cells was described in healthy individuals and patients with OC [223]. In addition, DC-loaded Nanog-specific peptides with the ability to induce a strong anti-tumour immune response against CSCs have been proposed [224]. These approaches may evoke immunological memory with the ability of suppressing CSC propagation in the recurrence scenario. These observations require future studies to develop CSC-associated immunotherapy. This is especially important in the scenario of OC dissemination where downregulation of HLA class 1 antigen, interferon induced pathway and upregulation of immune inhibitory PD-L1 is evident on tumour cells after chemotherapy treatment [123].

#### Signaling Pathways as Potential Targets for Ovarian CSCs 

As mentioned before, several signaling pathways collaborate for the initiation and sustenance of ovarian CSCs. These have been discussed extensively in a recent review [86]. PI3K/PTEN/AKT inhibitors such as BKM120, Everolimus and Perifosine are being used to treat OC patients [80,225]. Our group has recently shown enhanced expression of STAT3 in ascites-derived recurrent tumour cells compared to chemonaive tumour cells by genomic and proteomic approaches [226]. Eighty-six% of ovarian tumours and 63% of HGSOC have constitutive pSTAT3 and tissue array of women with high constitutive pSTAT3 have shown poor survival compared to women with low constitutive pSTAT3 [227]. In addition, enhanced expression of pSTAT3 was noted in recurrent ovarian tumours compared to primary tumours [228]. In pre-clinical mouse models, the credibility of JAK2/STAT3 inhibitor Momelotinib in combination with paclitaxel has shown a potential in suppressing the expression of OC CSC markers leading to reduced tumour burden in a mouse model [81,82,83]. Association between stem cell factor Nanog and CD44 was shown to activate STAT3 pathway in OC cells [229]. This resulted in the expression of multidrug resistant gene and concurrent chemoresistance. Hence, JAK2/STAT3 specific inhibitors may have a crucial role in suppressing and/or eradicating CSC-related disease in OC patients. In addition, inhibitory agents that target critical steps in the Wnt, Notch, and Hh pathways are currently in clinical development [86,88]. The Hh inhibitor GDC-0449 is currently in clinical trial for OC patients [88]. Cyclopamine (LDE 225, a naturally occurring alkaloid found in corn lily) was shown to reverse taxane resistance in OC cell lines [230].

### 6.10. Metabolic Targeting of Ovarian CSCs

The metabolic profile of OC cells and ovarian CSCs has been described in a recent review [123]. It is becoming obvious that ovarian CSCs sustain a malleable metabolism, which depends on their nutritional requirement and the stimulus they receive from the tumour microenvironment [231]. Reprograming of metabolism from glycolysis to tricarboxylic acid (TCA) cycle to an active lipid metabolism was noted in CD44^+^CD117^+^ enriched primary culture grown as a suspension culture [232]. On the other hand, CSC-like spheroid derived in vitro from OC cells and primary tumours showed dependency on anaerobic glycolysis and pentose phosphate pathway (PPP) [233]. PPP pathway in this scenario was essential for NADPH synthesis to facilitate fatty acid synthesis, which are essential nutrients for floating anchorage independent cells [233]. A recent study showed a significant enhanced level of unsaturated lipids in ovarian CSCs compared to non-CSCs [234]. High lipid unsaturation level was also noted in spheroids, which were enriched with CSCs compared to monolayer cultures of OC cell lines or primary cultures [234]. Inhibition of lipid desaturases eliminated CSCs, suppressed sphere formation in vitro, and suppressed tumour development in vivo [234]. Consistent with that, we have recently shown that chemotherapy-resistant recurrent OC cells from ascites of OC patients exhibit features of CSCs, and demonstrate OXPHOS-dependent acetyl-CoA-driven lipid metabolism [123]. This is also consistent with the most recent observation in which complete eradication of OC dissemination was achieved by targeting lipid metabolism with a metabolic inhibitor cocktail [235]. It should be noted that lipid metabolism via fatty acid oxidation is controlled by JAK2/STAT3 pathway [236] which we previously have shown supports chemotherapy surviving ovarian CSCs [81,82,83].

### 6.11. Role of Magmas Inhibitor in Ovarian Cancer Treatment

Due to metabolic and signaling irregularities, cancer cells produce high levels of ROS, which facilitate disease progression by generating further mutations and stimulating oncogenes [237]. Excessive production of ROS in cancer is oncogenic and can promote DNA damage, aggressiveness, resistant to therapy, genetic instability, immune dysregulation, cell death or senescence [238,239]. However, recent studies have indicated that increased level of antioxidants are also prevalent in cancer cells to detoxify ROS and at the same time sustain enhanced mitogenic signaling to promote tumourigenesis, resistance to therapy and apoptosis [240]. Increased ROS production has been reported for ovarian tumours [241]. This may relate to genetic instability, DNA damage repair deficiency, enhanced aggressiveness and the chemoresistant phenotype of the disease as discussed previously. In addition, ovarian tumours develop and reside in a harsh hypoxic peritoneal microenvironment, which is characterized by low oxygen tension [242]. The presence of ascites in 40% of the HGSOC further aggravates the hypoxia-mediated condition as ascites has 50% less soluble oxygen compared to normal blood [243]. In response to low oxygen hypoxia-inducible transcription factor-1 alpha (HIF-1α) gathers and binds to the hypoxia-response elements of different target genes, enhancing the activation of genes associated with adaptive survival, apoptosis, tumour cell migration, invasion, angiogenesis, etc. [244]. Under hypoxia the intracellular levels of ROS increases which is mediated through the complex III of the mitochondrial electron transport chain [245]. ROS stabilizes and activates HIF-1α that leads to transcriptional induction of genes related to tumour progression [245,246]. A recent study has shown that nuclear expression of HIF-1α is an independent bad prognostic marker of OC [247]. Another study identified the ROS levels in the tumours of 34 HGSOC patients and found that it was eight times higher in the tumours of Stage III/IV patients compared to normal ovaries [248]. As Magmas has a distinct role as a scavenger of ROS [119], it is not surprising that a high expression of Magmas was seen in a HGSOC compared to benign serous tumours (Figure 1). Considering the studies described above it can be postulated that high expression of Magmas in HGSOC is related to high ROS levels due to high HIF-1α expression in HGSOC. Enhanced Magmas expression under this scenario may be required to detoxify increased levels of ROS but at the same time maintain enhanced mitogenic and tumourigenic signals. Consistent with that, OC cell lines also demonstrate mitochondrial staining of Magmas (Figure 2). We also demonstrate that Magmas mRNA expression is enhanced in ovarian cancer cell lines in response to chemotherapy treatments, and that enhanced Magmas expression coincides with enhancement in the mRNA expression of drug-resistant gene ERCC1 and embryonic stem cell marker Oct4 (Figure 3). Consistent with that, we show that Magmas expression is enhanced in mouse xenografts obtained after treatment with paclitaxel compared to control untreated mice (Figure 4). In addition, enhanced sensitivity of carboplatin-resistant OV90 cell line to Magmas inhibitor, BT#9, compared to parental cell line (Figure 5) again confirms a role for Magmas in drug resistance. In this context, platinum-based chemotherapies have been shown to induce increased oxidative stress in cancer cells [249]. In addition, we have previously shown that platinum-based drugs readily induce EMT phenomenon in OC cells [76,78]. A recent study on breast cancer stem cells (BCSCs) demonstrated that metabolic and oxidative stress transitions the ROS^low^ mesenchymal BCSCs to a ROS^high^ epithelial state [250]. The increased response of carboplatin-resistant OV90 cell line to BT#9 compared to parental control is under investigation. However, it can be postulated that prolonged treatment with carboplatin in resistant OV90 cells may have induced EMT making them ROS^low^ mesenchymal cells, which are readily labile to BT#9. This is consistent with the recent observation that hypoxia-induced ROS production in platinum-resistance OC cells undergoes increase in mitochondrial fission [251]. It was shown that the scavenging of ROS by N-acetyl cysteine and Trolox could abolish this response of mitochondrial fission [251]. Hence, targeting mitochondrial protein Magmas, which regulates ROS production, could potentially be used as an anti-OC therapy.

OC patients initially respond to a first-line of chemotherapy treatment (~80% of patients respond to therapy). However, most patients eventually relapse with chemoresistant disease. The development of chemoresistance-associated recurrence is a major cause of poor prognosis in OC patients (5-year survival of less than 30% in most cases). The greater toxicity effect of BT#9 towards carboplatin resistant OC cells may add a new dimension to the treatment of chemoresistant OC patients.

## 7. Conclusions

HGSOC is a deadliest form of cancer due to its widespread peritoneal dissemination at diagnosis and frequent episodes of recurrences, which consequently results in peritoneal organ failure leading to patient’s morbidity. Ovarian CSCs, which form a small sub-population of the tumour, resist conventional therapies and are responsible for recurrence and extensive peritoneal dissemination post-chemotherapy treatments. However, the plasticity of CSCs and their inherent heterogeneity based on the expression of different markers makes them somewhat difficult to target. Hence, to address this challenge there is a need to discover putative agents, which will target CSCs in combination with chemotherapy so that the enrichment of CSCs by chemotherapy treatment is deterred. The observations that Magmas is overexpressed in HGSOC, is associated with chemotherapy resistance and CSCs suggests that Magmas have a role in chemotherapy resistance. The fact that Magmas specific inhibitor BT#9 has a significant inhibitory effect on glioma-stem cells suggests that designing future treatment strategies for OC specific CSCs with BT#9 may be promising. It can be postulated that BT#9 may have the same effect on ovarian CSCs as glioma stem cells and may be effective in decreasing ovarian recurrence. In addition, our preliminary data suggests that BT#9 has greater cytotoxic effects on carboplatin resistant OV90 cell line compared to non-resistant parental cell line reinforces that the development of Magmas-based therapeutics may have a strong potential for better treatment outcome in OC patients. Validation of this concept will introduce a new paradigm to the clinical management of OC patients.

## Figures and Tables

**Table 1:** List of clinical trials (ongoing and closed) described in the review involving PARP Inhibitors, Antiangiogenic Agents/Chemotherapy and Immune Checkpoint Inhibitors.

PARP Inhibitors	PARP Inhibitors + Antiangiogenic Agents/Chemotherapy	Immune Checkpoint Inhibitors in Combination with Other Anti-Cancer Agents
Gelmon KA et al.: Olaparib in patients with recurrent high-grade serous or poorly differentiated ovarian carcinoma or triple-negative breast cancer: a phase 2, multicentre, open-label, non-randomised study [136].	Coleman RL et al.: Veliparib with First-Line Chemotherapy and as Maintenance Therapy in Ovarian Cancer [147].	Burger R et al.: NRG Oncology phase II trial of nivolumab with or without ipilimumab in patients with persistent or recurrent ovarian cancer. 17^th^Biennial meeting of the International Gynecologic Cancer Society, September 14–16, 2018, Kyoto, Japan [171].
Ledermann J et al.: Olaparib maintenance therapy in platinum-sensitive relapsed ovarian cancer [137].	Mirza MR, et al.: Combination of niraparib and bevacizumab versus niraparib alone as treatment of recurrent platinum-sensitive ovarian cancer. A randomized controlled chemotherapy-free study-NSGO-AVANOVA2/ENGOT-OV24 [148].	Wenham RM etal: Phase 2 trial of weekly paclitaxel with pembrolizumab in platinum recurrent ovarian cancer. 17^th^Biennial meeting of the International Gynecologic Cancer Society, September 14–16, 2018, Kyoto, Japan [172].
Swisher EM et al.: Rucaparib in relapsed, platinum-sensitive high-grade ovarian carcinoma (ARIEL2 Part 1): an international, multicentre, open-label, phase 2 trial [138].		Grunewald CM et al: Tumor immunotherapy-the potential of epigenetic drugs to overcome resistance. Translational Cancer Research 2018, 1151–1156. [173].
Mirza MR, et al.: Niraparib Maintenance Therapy in Platinum-Sensitive, Recurrent Ovarian Cancer [139].		
Oza AM et al.: Quality of life in patients with recurrent ovarian cancer treated with niraparib versus placebo (ENGOT-OV16/NOVA): results from a double-blind, phase 3, randomised controlled trial [140].		
Friedlander M et al.: Health-related quality of life and patient-centred outcomes with olaparib maintenance after chemotherapy in patients with platinum-sensitive, relapsed ovarian cancer and a BRCA1/2 mutation (SOLO2/ENGOT Ov-21): a placebo-controlled, phase 3 randomised trial [141].		
Ledermann J et al.: Olaparib maintenance therapy in patients with platinum-sensitive relapsed serous ovarian cancer: a preplanned retrospective analysis of outcomes by BRCA status in a randomised phase 2 trial [142].		
Pujade-Lauraine E et al.: Olaparib tablets as maintenance therapy in patients with platinum-sensitive, relapsed ovarian cancer and a BRCA1/2 mutation (SOLO2/ENGOT-Ov21): a double-blind, randomised, placebo-controlled, phase 3 trial [143].		
Coleman RL et al.: Rucaparib maintenance treatment for recurrent ovarian carcinoma after response to platinum therapy (ARIEL3): a randomised, double-blind, placebo-controlled, phase 3 trial [144].		
Liu JF et al.: Combination cediranib and olaparib versus olaparib alone for women with recurrent platinum-sensitive ovarian cancer: a randomised phase 2 study [145].		
Ledermann JA et al.: Overall survival in patients with platinum-sensitive recurrent serous ovarian cancer receiving olaparib maintenance monotherapy: an updated analysis from a randomised, placebo-controlled, double-blind, phase 2 trial [146].		
